# miR‐302b regulates cell cycles by targeting CDK2 via ERK signaling pathway in gastric cancer

**DOI:** 10.1002/cam4.818

**Published:** 2016-07-27

**Authors:** Fu‐Yun Liu, Li‐Ping Wang, Qin Wang, Ping Han, Wen‐Ping Zhuang, Mu‐Juan Li, Hua Yuan

**Affiliations:** ^1^ Department of NursingLinyi People's HospitalLinyi276003China; ^2^ Department of Gastrointestinal SurgeryLinyi People's HospitalLinyi276003China; ^3^ Department of Gastrointestinal Surgerythe First Affiliated Hospital of Guangxi Medical UniversityNanning530021China

**Keywords:** CDK2, ERK, gastric cancer, miR‐302b

## Abstract

To investigate the molecular mechanism of miR‐302b in the regulation of cell proliferation and cell cycle regulation in gastric cancer. Samples of tumor and adjacent normal tissues were collected from 30 gastric cancer patients. Bioinformatics and the dual luciferase report were used for verification of the relationship between miR‐302b expression and cyclin‐dependent kinase 2 (CDK2). RT‐PCR and western blot were used to examine CDK2 mRNA and protein levels. The impacts of miR‐302b on CDK2 expression and extracellular signal‐regulated kinase (ERK) signaling pathway were assessed in cells transfected with miR‐302b analogs and CDK2 overexpression carrier, respectively. We used 3‐(4,5‐dimethylthiazol‐2‐yl)‐2, 5‐diphenyltetrazolium bromide (MTT) to assay gastric cancer cell growth after transfection, flow cytometry to analyze cell cycle. Compared with normal tissues, miR‐302b expression was significantly lower in gastric cancer tissues, which was significantly related to lymph node metastasis, metastasis distance, and TNM staging. miR‐302b expression was increased in miR‐302b mimics transfected cells and was significantly decreased in miR‐302b inhibitors transfected cells. CDK2 is a target gene of miR‐302b. Decreased miR‐302b and increased CDK2 expressions can significantly promote proliferation and G1/S phase transformation in gastric cancer. miR‐302b promoted the proliferation of gastric cancer cells through upregulation of CDK2, thereby inhibiting ERK pathway, which can in turn inhibit the promoting ability of miR‐302b on proliferation. The upregulation of miR‐302b reduced the expression of CDK2, and inhibited ERK signaling pathway, thereby inhibiting cell proliferation and G1/S phase conversion rate. Therefore, miR‐302b provides new perspectives for research of cell regulation and proliferation in gastric cancer, and new targets for gastric cancer diagnosis and treatment.

## Introduction

Gastric cancer is the most common form of gastrointestinal cancer. A survey in 2008 found that gastric cancer accounted for 8% of all new cancer cases, and 10% of all cancer mortalities worldwide 1. Data showed that the incidence and mortality rate of gastric cancer were higher in China than the global average, with the diagnosis rate of approximately 10% for early‐stage cancer, and 90% for advanced cancer. The 5‐year survival rate of early gastric cancer was more than 70%, but <10% for advanced gastric cancer 2, 3, 4. Therefore, early diagnosis and treatment have great merit in lowering both incidence and mortality rates of gastric cancer. The onset of gastric cancer is considered to be a combination of genetic factors, environmental factors, and lifestyle, of which the exact mechanism remains unclear 5, 6_._ In recent years, we found that MicroRNA (miRNA)‐mediated transcriptional regulation was related to gastric cancer pathogenesis, which may provide new perspective for researches on the pathogenesis and early diagnosis of gastric cancer 7, 8.

miRNA is a class of 19‐25 nt noncoding single‐stranded small molecule RNA commonly found in plants and animals. miRNA can inhibit transcription and translation of a target gene via incomplete binding to target miRNA 9. It is reported that about two‐thirds of miRNA can regulate the expression of human proteins, which are associated with ontogeny, cell proliferation, differentiation, and apoptosis 10. It has been confirmed that about 50% miRNA that have been studied are positioned on cancer‐related chromosomal fragile sites, and the cleavage sites of cancer genes or cancer‐suppressing genes 11, 12. miRNA is also related to gastric cancer proliferation and metastasis. The cyclin‐dependent kinase 2 (CDK2) gene is a type of cell cycle protein kinases, whose activation causes malignant cell proliferation 13. Previous studies suggested that CDK2 was a positive regulator of gastric cancer cell cycle, which can be abnormally activated by increased degrees of malignancy and the invasion of cancer cells 14. Extracellular signal‐regulated kinase (ERK) belongs to the mitogen‐activated protein kinase (MAPK) family. The ERK signaling pathway involves in many cell proliferation and differentiation processes, and has been found to be related with the invasion and metastasis mechanisms in multiple cancers 15. However, a relationship between miR‐302b and the ERK signaling pathway has not been reported. This study aims to evaluate the relationship between miR‐302b and ERK signaling pathway, and the underlying molecular mechanisms by examining the expression of miR‐302b and CDK2, and their roles in gastric cancer cells.

## Materials and Methods

### Tissue source

Tissue sample were collected from tumor tissue and the adjacent normal tissues (about 10 cm from the tumor tissue) of 30 patients with pathologically confirmed gastric cancer and received surgery, during January 2009 to December 2013 at our hospital. All patients underwent gastroscopy check and had their cancer confirmed by pathology examination. The patients did not receive preoperative radiotherapy or chemotherapy, were free of inflammation and other complications, and their corresponding normal gastric tissues were not affected. Each specimen was examined and diagnosed by two or more physician independently. Of the participants, 18 were male and 12 were female; 20 cases aged >60 years, and 10 cases were <_60 years old; the average age was 58.6 ± 16.9 years; 16 cases had well‐differentiated carcinoma, and 14 cases had poorly differentiated carcinoma. Tumor stage was determined using the TNM staging criteria according to the 2010 American Joint Committee on Cancer (AJCC) Guidelines on TNM staging of gastric cancer 16, 13 cases were stage I/II gastric cancer, 17 cases were stage III/IV; 15 cases did not have lymph node metastasis, and 15 cases had lymph node metastasis. The inclusion criteria for each eligible patient were as follows: (1) patients who were initially and pathologically diagnosed with gastric cancer; (2) no radiotherapy and chemotherapy or biological therapy was conducted prior to diagnose and surgery; (3) complete medical information was required; (4) no other complications. Meanwhile, the exclusion criteria were displayed: (1) patients with other malignant tumor of digestive tract; (2) patients who were unlikely to tolerate gastroscopy; (3) female patients during pregnant stage and breast‐feed stage. All samples were obtained during surgery, and were quickly preserved using liquid nitrogen. The experimental protocol was approval by the Ethics Committee of our hospital. All patients were informed and signed the consent form. All procedures in this study were in compliance with the Declaration of Helsinki 17.

### Cell culture and cell transfection

Normal gastric cell line GES‐1, gastric cancer cell line SGC‐7901, MGC‐803, MKN‐28, and BGC‐823 (all purchased from American Type Culture Collection, ATCC) were cultured with 10% fetal calf serum volume fraction of RPMI‐1640 in 5% CO_2_, 37°C, humidity 95% CO_2_ incubator. Cultured cells were grown as monolayers, with a cell attachment rate of 90% or more. For passage, the medium was drained, and the culture was washed with phosphate‐buffered saline (PBS) twice and digested with 0.25% trypsin. When cell gap increased, the trypsin was decanted and cells were pipetted with 10% RPMI‐1640 medium calf serum into a single cell suspension for routine passage. Cells in the logarithmic growth phase were used for subsequent research.

Experimental groups: (1) miR‐302b mimics group (transfected with synthetic miR‐302b analogs, miR‐302b analogs and a miR‐302b inhibitor purchased from Shanghai Jima biotech company); (2) miR‐302b‐NC group (transfected with nonsense sequence, abbreviated as NC group); (3) miR‐302b inhibitors (transfected with a miR‐302b inhibitor); (4) Mock group (control, not transfected), transfected sequences are shown in Table 1. A day before the transfection, an appropriate number of cells were inoculated in a 6‐well culture plates with 2 mL of an antibiotic‐free medium to reach a cell density of 50–60% at the time of transfection. After removing the old medium, the culture was washed twice with serum‐free Opti‐MEM I medium and 11.5 mL of Opti‐MEM was added to each well. Then, 250 *μ*L of serum‐free culture medium Opti‐MEM I was used to dilute 5 *μ*L of mimics, inhibitors, and NC. RNA was added to each group with a final concentration of 50 nmol/L, followed by gentle mixing and incubation at room temperature for 5 min. The diluted Lipofectamine 2000 (Invitrogen, Carlsbad, CA, USA) and the diluted mixture were then gently transfected and incubated at room temperature for 20 min. The prepared transfection reagent mixture was added to each well at 500 *μ*L/well and mixed gently. The plates were incubated at 37°C in 5% CO_2_ incubator for 6 h. After 6 h, the old medium was replaced by a fresh Dulbecco's minimum essential medium (DMEM) containing 10% fetal bovine serum. Then the culture was placed under the laser confocal microscope at a wavelength of cy3 to capture images.

**Table 1:** Transfected sequence

Gene	Primer sequences
Mimics	5′‐AUUCACGAAGGUACAAAAGCAU‐3′
Inhibitors	5′‐TACGAAAACATGGAAGCACTTA‐3′
NC	5′‐UGUGGUUAUAUGUAACAUCCUU‐3′
	5′‐ACACCAAUAUACAUUGUAGGAA‐3′

NC, negative control.

### Dual luciferase report analysis

Bioinformatics was used to predict the presence of a close relationship between the complementary bases miR‐302b and CDK2, and the expression vector of the seed region of mutant CDK2. The SGC‐7901 cells were seeded in 96‐well plates, about 4 × 10^3^ cells/well, which was then divided into four groups and transfected with miR‐302b mimics + CDK2‐WT (WT + mimics group), miR‐302b mimics + CDK2‐MT (MT + mimics group), miR‐302b NC + CDK2‐WT (WT + NC group), miR‐302b NC + CDK2‐MT (MT + NC group), respectively. The culture medium was replaced after 6 h, and the SGC‐7901 cells were collected at 72 h after transfection. Then, cell fluorescence signal and Renilla luciferase signal were measured by fluorescence light detector.

### Real‐time polymerase chain reaction (RT‐PCR) assessment of miR‐302b and CDK2 mRNA expression

Frozen tissue (50 mg) and cells from each group after transfection were used to extract the total RNA using Qiagen's miRNeasy Mini Kit (Qiagen, Hilden, Germany). A 5 *μ*L of RNA sample was diluted 20‐fold by ultrapure water without RNA enzyme, and measure for absorbance at 260 nm and 280 nm in the UV spectrophotometer. The purity of RNA measured as the OD260/OD280 ratio was between 1.7 and 2.1, which indicated a high purity that met research needs in the subsequent experiments. In the PCR amplification reaction, RT cDNA was used as a synthesis template. Then, real‐time quantitative RT‐PCR was performed using ABI7500 under the following conditions: predenaturation at 95°C 10 min, denaturation at 95°C for 10 sec, annealing at 60°C for 20 sec, extension at 72°C for 34 sec, repeated for 40 cycles. The amplification primer sequences are shown in Table 2. The CDK2 fluorescence quantitative PCR reaction volume was 50 *μ*L, including cDNA template 5.0 *μ*L, upstream and downstream of each primer 0.5 *μ*L, 10 × buffer 25.0 *μ*L, 2 × SYBR Green q PCR Super Mix 10.0 *μ*L, ddH_2_O 4.0 *μ*L. The reaction conditions were as follows: denaturation at 94°C for 2 min, 94°C for 30 sec, annealing at 53°C for 30 sec, extension at 72°C for 5 min; total reaction was amplified for 40 cycles. The primer sequences are shown in Table 2. The thresholds were manually selected at the lowest point of each amplification curve to obtain threshold cycle (Ct) values of the reaction tube. Data were analyzed using 2^−ΔΔCt^ method, 2^−ΔΔCt^ represents ratios of gene expression between the experimental group and the control group, expressed as the following formula: ΔΔCt = [Ct_(target gene)_ – Ct_(reference gene)_]_experimental group_ − [Ct_(target gene)_−Ct_(reference gene)_]_the control group_. This experiment was repeated three times.

**Table 2:** Gene primer sequences

Gene	Primer sequences
miR‐302b	F: 5′‐ ATCCAGTGCGTGTCGTG‐3′
	R: 5′‐ TGCTTAAGTGCTTCCATGTT‐3′
U6	F: 5′‐ TGCGGGTGCTCGCTTCGGCAGC‐3′
	R: 5′‐ CCAGTGCAGGGTCCGAGGT‐3′
CDK2	F: 5′‐GCGAATTCCCCAGCCCTAATCTCA‐’3
	R: 5′‐GCCTCGAGAACCCTCTTCAGCAATAA‐’3
GAPDH	F: 5′‐ACACCATGGGGAAGGTGAAG‐’3
	R: 5′‐AAGGGGTCATTGATGGCAAC‐’3

F, forward; R, reverse. CDK2, cyclin‐dependent kinase 2

### Western blot assessment of the expression of CDK2, ERK, pERK protein in transfected cells

Protein samples were total protein collected from the transfected groups, and samples containing the target protein were separated by sodium dodecyl sulfate polyacrylamide gel electrophoresis (SDS‐PAGE), and electrophoretic transfer to polyvinylidene difluoride (PVDF) membrane surface in situ. After the transfer, the PVDF membrane was transferred to the blocking solution and mixed well with 1: 1000 dilution of rabbit anti‐human CDK2, ERK, pERK antibodies (purchased from GeneTex, Inc., San Antonio, TX), and incubated overnight at 4°C. The membrane was washed three times with 1 × TBST for 10 min at room temperature and then mixed with a secondary antibody (goat anti‐rabbit 1: 500; purchased from Shanghai Beizhuo Biological Technology Corporation), and incubated for 1 h. The positive side of the PVDF membrane was contacted for 3–5 min with the working fluid used to prepare electrochemiluminescence (ECL) liquid, drained of the ECL solution and transferred to the darkroom for developing images, captured by the ImageMaster VDS photographed image analysis system (Image Master Compact HR, Trioptics Co., Germany). Each set of samples underwent three examinations. We used glyceraldehyde phosphate dehydrogenase (GAPDH) as an internal reference.

### MTT colorimetric test

When 80% of the transfected cells accrued, they were washed twice using PBS solution. Cells were trypsinized and pipetted into single cell suspension and counted using a cytometer. Cells were seeded separately in six wells on a 96‐well plate. Each well was inoculated with 3 × 10^3^–6 × 10^3^ cells, with the volume of each well to being 200 *μ*L. The cells were incubated at 37°C, in 5% CO_2_ incubator for 24–96 h. Then, 20 *μ*L of the MTT (5 mg/mL, Sigma Chemical Co., St. Louis, MO) solution was added for coloration. Continuing incubation at 37°C in 5% CO_2_ incubator for 4 h until the termination of the culture, after which the medium was discarded. Then, 150 *μ*L of dimethyl sulphoxide (DMSO) was added to each well and then the wells were shaken lightly for 10 min to foster the crystals to be dissolved. The absorbance of each well was assessed using enzyme‐linked immunosorbent assay (ELISA) reader at 12 h, 24 h, 48 h, and 72 h, and plotted the MTT curve with the absorbance value as the *y*‐axis, and the time interval as the *x*‐axis. The procedure was repeated three times.

### Flow cytometry assessment of cell cycle

The cells were seeded in 6‐well culture plates with 1 × 10^6^ cells in each well, and cultured simultaneously for 12 h. After removing the original medium, cell cultures were digested, cells were collected by centrifugation, washed twice with PBS, resuspended with precooled 75% ethanol, and fixed overnight at −20°C, then centrifuged with the supernatant discarded. The cells were then washed twice by PBS, and 450 *μ*L PBS resuspended cells were taken from each sample. The collected cells were added with 50 *μ*L of propidium iodide (PI: 0.5 mg/mL), mixed and transferred to 37°C water bath for 30 min, and centrifuged to remove the supernatant. The cells were resuspended with PBS again and measured for cell cycle distribution by a flow cytometer (FACSCalibur; BD Biosciences, Mountain View, CA).

### Statistical analysis

All data were analyzed using SPSS 21.0 statistical software (SPSS Inc., Chicago, IL, USA). Data were presented as mean ± standard deviation (SD) of three experiments. Analysis of variance (ANOVA) was used for comparison between multiple groups, and *t* test for comparison between two groups. *P *<* *0.05 indicates statistically significant difference.

## Results

### miR‐302b was significantly downregulated in gastric cancer tissues

**Figure 1:**
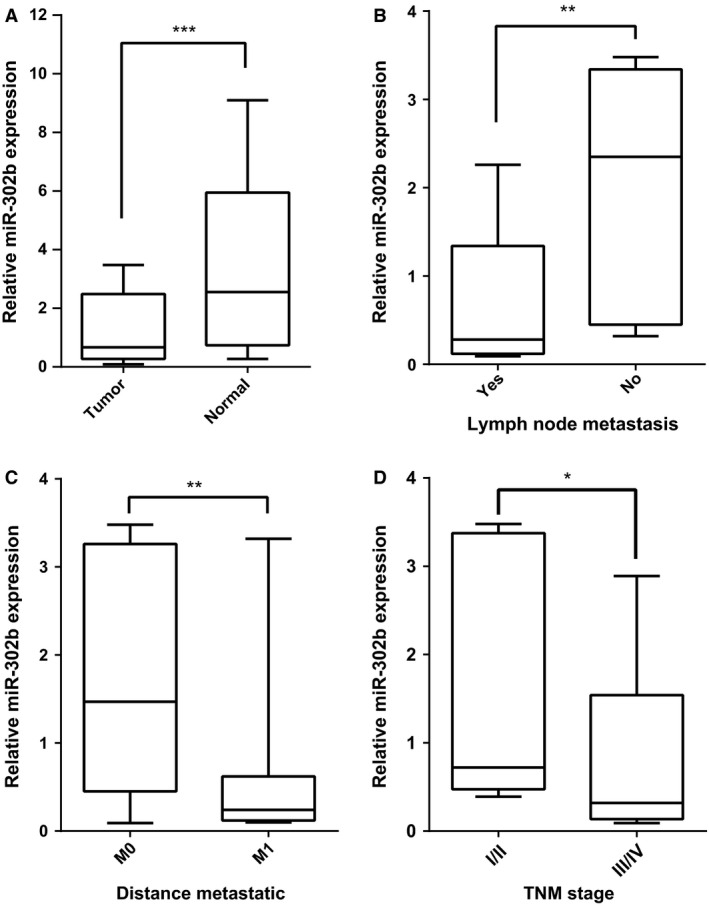
Real‐time polymerase chain reaction (RT‐PCR) assessment of miR‐302b expression levels. (A) miR‐302b expression is downregulated in human gastric cancer tissues relative to normal tissues; (B) miR‐302b expression level was significantly lower in gastric cancer patients with lymph node metastasis than that of patients without lymph node metastasis; (C) miR‐302b expression level was significantly lower in patients with a transfer distance of M1 than patients with transfer distance of M0; (D) miR‐302b expression was significantly lower in TNM III‐IV patients than in TNM I/II patients. ***P <* *0.0001; **P <* *0.01; *P <* *0.05.

The expression level (ΔCT) of miR‐302b was significantly reduced in gastric cancer tissue compared to that of the adjacent normal gastric tissues (*P *<* *0.01) (Fig. 1A). Further analysis of the association between miR‐302b expression level and clinical characteristics showed that miR‐302b expression level in gastric cancer patients with lymph node metastasis (0.28 [0.15–1.34]) was significantly lower than that of patients without lymph node metastasis (2.35 [0.45–3.34]), the difference was statistically significant (*P *=* *0.006). The expression levels were significantly lower in patients with a metastasis distance of M1 (0.24 [0.12–0.62]) than patients with a metastasis distance of M0 (1.47 [0.42–3.26]), the difference was statistically significant (*P *=* *0.005). The expression level was significantly lower in TNM III–IV patients (0.32 [0.15–1.47]) than in TNM I/II patients (0.72 [0.5–3.41]), the difference was statistically significant (*P *=* *0.023) (Fig. 1B, C and D); but the expression of miR‐302b was not associated with gender, age, disease location, degree of differentiation, or depth of invasion (all *P > *0 .05) (Table 3).

**Table 3:** Correlation between miR‐302b and clinical parameters in patients with gastric cancer

Clinical features	*N*	The relative expression of miR‐302b	*Z, H* value	*P* value a
Sex				
Male	18	0.50 (0.28–2.35)	−0.296	0.767
Female	12	0.72 (0.24–3.32)		
Age (years)				
>60	20	0.45 (0.24–2.26)	−0.264	0.792
<_60	10	0.62 (0.11–2.35)		
Tumor location				
Upper abdomen	8	0.50 (0.15–3.45)	0.486	0.784
In the abdomen	9	0.51 (0.12–3.46)		
Lower abdomen	13	0.72 (0.32–2.26)		
Differentiation				
Poor	14	0.78 (0.39–2.89)	−1.455	0.146
Medium	16	0.5 (0.15–1.61)		
Lymph node metastasis				
Yes	15	0.28 (0.15–1.34)	−2.758	0.006
No	15	2.35 (0.45–3.34)		
Distant metastasis				
M0	19	1.47 (0.42–3.26)	−2.819	0.005
M1	11	0.24 (0.12–0.62)		
Depth of invasion				
T1 + T2	12	0.42 (0.24–3.26)	−0.064	0.949
T3 + T4	18	1.61 (0.62–1.61)		
TNM staging				
I/II	13	0.72 (0.5–3.41)	−2.281	0.023
III/IV	17	0.32 (0.15–1.47)		

Relative expression level was represented by the median and the upper and lower quartiles in parenthesis.

a *P *<* *0.05 indicates significant differences (using the Mann–Whitney *U* test between two groups, and Kruskall–Wallis test between the three groups).

### miR‐302b expression in exogenous upregulated or blocked gastric cancer cell lines

**Figure 2:**
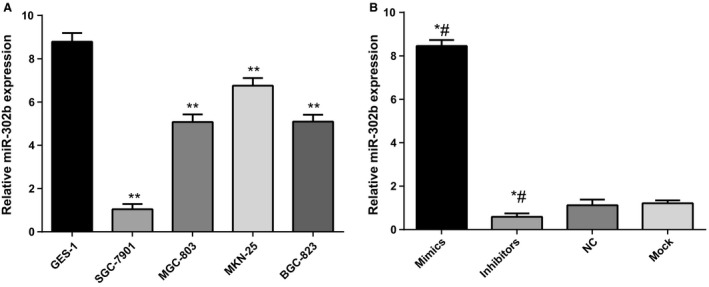
miR‐302b expressions in exogenously upregulated or blocked gastric cancer cell lines. (A) miR‐302b expression in gastric cancer cell lines. **compared with GES‐1 cells, *P *<* *0.0001. (B) miR‐302b expressions in exogenously upregulated or blocked gastric cancer cell lines. *compared with NC group, *P *<* *0.0001; #compared with Mock group, *P *<* *0.0001.

The miR‐302b expression levels were assessed in four gastric cancer cell lines SGC‐79 01, MGC‐803, MKN‐25, BGC‐823, and normal gastric epithelial cell GES‐1 by real‐time quantitative PCR. Quantitative results showed that miR‐302b expression in SGC‐7901, MGC‐803, MKN‐25, and BGC‐823 cell lines was significantly lower than that in GES‐1 cells (8.7878 ± 0.4010) (all *P *<* *0.0001), and SGC‐7901 cells had the lowest expression level (1.0468 ± 0.2407; *P *<* *0.0001), therefore, we used SGC‐7901 cell line as mode cells to investigate the function of miR‐302b (Fig. 2A). Quantitative PCR of transfection efficiency showed the expression of miR‐302b was increased in cells transfected with miR‐302b mimics (*P *<* *0.0001), but decreased in cells transfected with miR‐302b inhibitors (*P *<* *0.0001) (Fig. 2B). These results confirmed that the exogenous upregulation or blockage of miR‐302b expression showed significant effect.

### The influence of miR‐302b on cell proliferation and cycle

**Figure 3:**
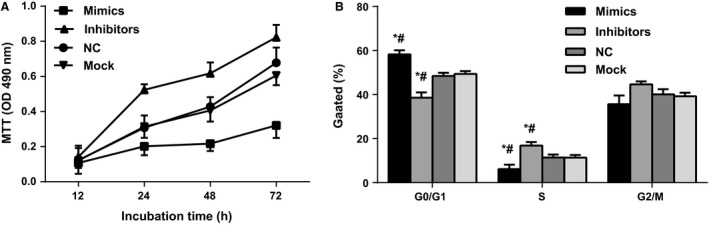
Influence of miR‐302b on cell proliferation and cycle. (A) MTT assays were performed to determine the proliferation of SGC‐7901cells; (B) The bar chart represents the percentage of cells in G0/G1, S, or G2/M phase, as indicated, *compared with NC group, # represents compared with Mock group. *P <* *0.001; #*P *<* *0.001.

In SGC‐7901 cells transfected with miR‐302b of inhibitors, mimics, NC, and Mock groups, MTT assay showed that cell proliferation activity was significantly enhanced in cells transfected with inhibitors (*P *<* *0.05), whereas the proliferation activity was significantly inhibited in cells transfected with mimics (*P *<* *0.001). The NC group and Mock group showed no significant difference (all *P > *0.05) (Fig. 3A). Further detection by flow cytometer showed G0/G1 phase cell proportion decreased significantly in the inhibitors group ([38.5366 ± 2.4401]%) compared with NC group ([48.4333 ± 1.4829]%) and Mock group ([49.3700 ± 1.2350]%) (both *P *<* *0.001), whereas the G0/G1 phase proportion increased in cells transfected with mimics ([58.2233 ± 1.8750]%) (*P *<* *0.001) while no significant difference was detected between NC group and Mock group (*P *>* *0.05) (Fig. 3B). These results suggest that downregulation of the expression of miR‐302b can significantly promote the proliferation of gastric cancer cells by promoting the conversion of the G1/S phase.

### CDK2 as a target gene of miR‐302b

**Figure 4:**
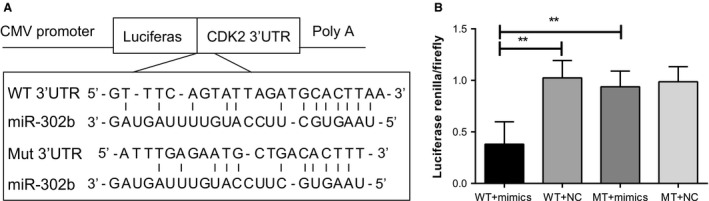
miR‐302b directly targets CDK2 by interacting with its 3′ UTR. (A) A human CDK2 3′UTR fragment containing wild‐type or mutant miR‐302b binding sequence was cloned downstream of the luciferase reporter gene. (B) The luciferase reporter plasmid containing wild‐type or mutant CDK2 3′UTR was cotransfected into SGC‐7901 cells. Luciferase activity was determined using the dual luciferase assay and shown as the relative firefly activity normalized to Renilla activity. **P <* *0.001. CDK2, cyclin‐dependent kinase 2.

Bioinformatics analyses showed that CDK2 might be a target gene of miR‐302b. According to the results of bioinformatics analysis, we cloned fragments in the CDK2 mRNA 3′UTR region that can bind to miR‐302b seed sequence, and bound the fragment into dual luciferase report gene vectors 3′ end (Fig. 4A). According to the results of sequencing, SGC‐7901 cells were transfected with the luciferase vector and miR‐302b overexpression vector for 48 h before the cells were lysed and tested for luciferase activity, which showed that miR‐302b can significantly inhibit the reported gene activity for luciferase containing CDK2 mRNA 3′UTR region. WT + mimics group had relative fluorescence activities of 0.3812 ± 0.2164, which was significantly lower than 1.0241 ± 0.1687 in WT + NC group (*P *<* *0.001). The relative fluorescence activities for MT + mimics group were 0.9680 ± 0.1527, higher than that 0.3812 ± 0.2164 for WT + mimics group (*P *<* *0.001). Similar fluorescence activities were found between MT + mimics group and MT + NC group (0.9680 ± 0.1527 vs. 0.9870 ± 0.1462) (Fig. 4B). Dual luciferase reporter gene activity test results showed that when the 3′UTR of CDK2 transferred to vectors with the luciferase gene, adding miR‐302b can inhibit luciferase activity, and when mutation occurred at the binding site between CDK2 3′UTR and miR‐302b, luciferase activity was no longer inhibited. These results confirmed that miR‐302b can be combined with the 3′UTR of the CDK2 mRNA seed region, providing direct evidence regarding the target relationship between miR‐302b and CDK2.

### CDK2 overexpression in SGC‐7901 cells

**Figure 5:**
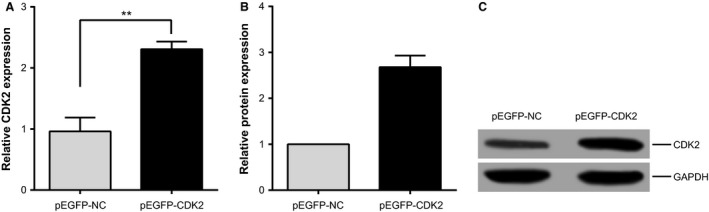
Overexpression of CDK2 in SGC‐7901 cells. (A) RT‐qPCR assay of CDK2 expression level, **P <* *0.0001. (B, C) Western blot assay of CDK2 protein levels; the mRNA and protein expression of SGC‐7901 cells were significantly elevated compared with SGC‐7901 cells transfected with empty plasmid (*P *<* *0.0001). CDK2, cyclin‐dependent kinase 2.

Clones of the full‐length CDK2 sequence were inserted into the eukaryotic expression vector pEGFP to construct the CDK2 overexpression vector pEGFP‐CDK2 (purchased from Shanghai Jima Biotech Companies), and was used to transfect SGC‐7901 cells. We used real‐time quantitative PCR and western blot technique were used to detect transfection efficiency and eukaryotic vector expression efficiency, and test results showed that the mRNA and protein expression for CDK2 in SGC‐7901 cells were, respectively, 2.3064 ± 0.1253 and 2.6784 ± 0.2514, both of which were significantly higher than that in the SGC‐7901 cells transfected with empty plasmid (*P *<* *0.0001) (Fig. 5A, B and C).

### Effect of CDK2 on gastric cancer cell proliferation and cycle

**Figure 6:**
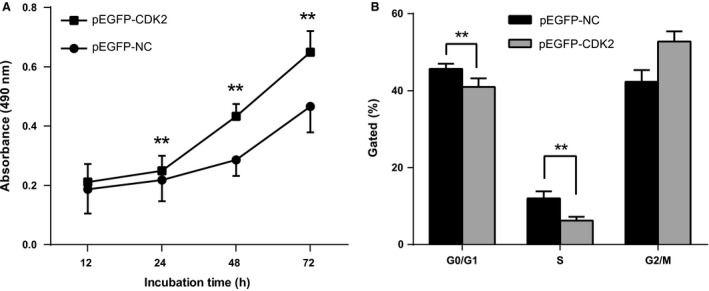
Influence of CDK2 on gastric cancer cell proliferation and cycle. (A) MTT assay of SGC‐7901 cell proliferation; (B) The histogram shows the percentage of the G0/G1, S, and G2/M phase cells. **P <* *0.0001; The bar chart represents the percentage of cells in G0/G1, S, or G2/M phase as indicated. **P <* *0.0001. CDK2, cyclin‐dependent kinase 2.

MTT and flow cytometry assay were used to test the influence of exogenous CDK2 upregulation on SGC‐7901 cell cycle progression and proliferation activity. Test results showed that SGC‐7901 cells transfected with pEGFP‐CDK2 with stable expression of CDK2 had significantly increased proliferation activities compared with cells transfected with empty vector (*P *<* *0.0001) (Fig. 6A). The proportion of G1 phase cells was significantly decreased, and the proportion of S phase cells was significantly increased (both *P *<* *0.0001) (Fig. 6B). These results indicate that increased CDK2 expression can significantly promote cell proliferation and G1/S phase conversion in gastric cancer cells.

### miR‐302b's oncogene function depends on CDK2 upregulation

**Figure 7:**
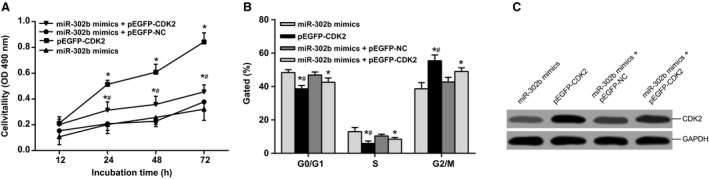
Inhibition of CDK2 is potentially involved in the oncogene function of miR‐302b. (A) MTT assay of SGC‐7901 cell proliferation; (B) The histogram shows the percentage of the G0/G1, S, and G2/M phase cells; (C) The expression of CDK2 protein was analyzed by western blot CDK2, cyclin‐dependent kinase 2.

The SGC‐7901 cells were divided into four groups and transfected with miR‐302b mimics, pEGFP‐CDK2, miR‐302b mimics + pEGFP – NC, and miR‐302b mimics + pEGFP‐CDK2, respectively (purchased from Shanghai Jima Biotechnology Companies), western blot assay of the expression of CDK2 protein showed that when miR‐302b mimics and pEGFP‐CDK2 were cotransfected, miR‐302b can inhibit the upregulation of CDK2 expression in single transfected pEGFP‐CDK2 (Fig. 7C). Furthermore, MTT and flow cytometry was used to show that cell proliferation in pEGFP‐CDK2 group and miR‐302b mimics + pEGFP‐CDK2 group were fast compared with miR‐302b mimics group and miR‐302b mimics + pEGFP – NC group. Moreover, a higher speed of cell proliferation was detected in pEGFP‐CDK2 group when compared with miR‐302b mimics + pEGFP‐CDK2 group (Fig. 7A–B). These results indicate that the influence of miR‐302b on the proliferation of gastric cancer cells is dependent on the increased expression of its target gene *CDK2*.

### Effect of miR‐302b‐induced CDK2 downregulation on the ERK pathway

**Figure 8:**
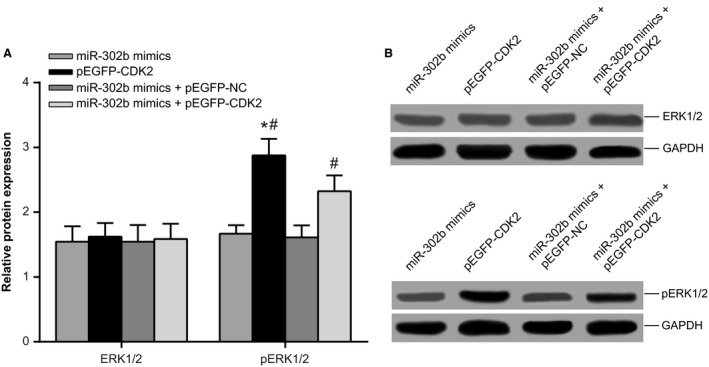
Western blot to assay the expression of ERK1/2 and pERK1/2 protein. Note: * Denotes comparison with miR‐302b mimics + pEGFP‐CDK2 group, *P *<* *0.05; # denotes comparison with miR‐302b mimics group and miR‐302b mimics + pEGFP – NC group, *P *<* *0.05. CDK2, cyclin‐dependent kinase 2; ERK, extracellular signal‐regulated kinase.

In the above transfected cell lines, we further assayed the expression of ERK1/2 protein and phosphorylated ERK1/2 (pERK1/2). The results showed that ERK1/2 protein levels did not significantly change in miR‐302b mimics, pEGFP‐CDK2, miR‐302b mimics + pEGFP – NC, or miR‐302b mimics + pEGFP‐CDK2 group (all *P > *0.05). Compared to the miR‐302b mimics + pEGFP‐CDK2 group, expression of pERK1 2 was increased in pEGFP‐CDK2 group (*P *<* *0.05), and decreased in the miR‐302b mimics and miR‐302b mimics + pEGFP – NC groups (both *P *<* *0.05), while no significant change was found in pERK1/2 protein expression among the miR‐302b mimics and miR‐302b mimics + pEGFP – NC groups (both *P > *0.05) (Fig. 8), suggesting that miR‐302b might induce CDK2 downregulation, thereby inhibiting the ERK pathway.

### Inhibition of the ERK signaling pathway can reverse miR‐302b's effect on gastric cancer cell proliferation

**Figure 9:**
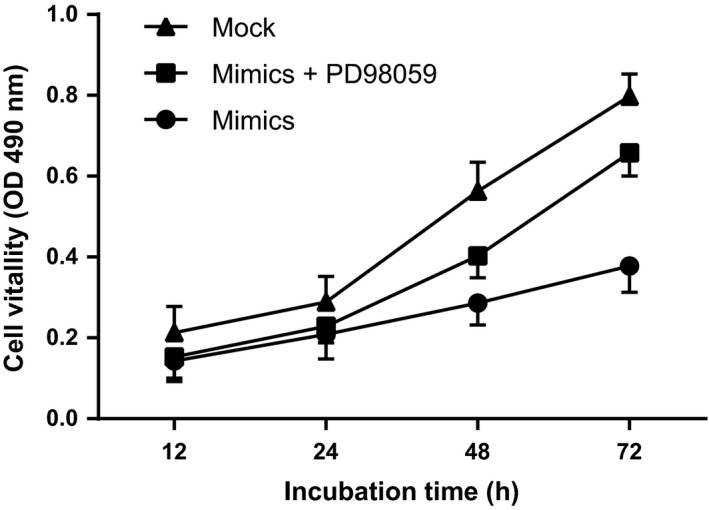
MTT of SGC‐7901 cell proliferation.

To further understand the role of the ERK signaling pathway in the miR‐302b‐induced promotion of SGC‐7901 cells proliferation, at the same time as miR‐302b overexpression, cells were treated with the ERK inhibitor PD98059 (purchased from New England Biolabs Inc., Beverly, MA.) to inhibit that miR‐302b‐induced ERK activation. The cells were then observed for changes in cell proliferation. The results are shown in Figure 9. The proliferation of SGC‐7901 cells decreased following the overexpression of miR‐302b, but the addition of PD98059 treatment lead to accelerated cell proliferation. These results suggest that the miR‐302b may promote gastric cancer cell proliferation mainly through the inhibition of the ERK signaling pathway.

## Discussion

Currently, the molecular mechanism that miRNA affects the expression of the gastric cancer cells, is not yet fully understood. Recent studies have shown that the miRNA regulation mechanism might be associated with DNA methylation and histone modifications. Many studies suggest that the genetic mutations and epigenetic regulation of miRNA expression induced upregulation or downregulation which plays an important role in the development of gastric cancer 18, 19, 20. In this study of the regulation and expression of miR‐302b, it was found that the cell cycle‐related proteins *CDK2* was a new target genes regulated by miR‐302b, and it appeared that miR‐302b‐mediated CDK2 upregulation could play an important role in the promotion of gastric cancer cell proliferation and cell phase conversion process.

Our results suggested that expression of miR‐302b was downregulated in gastric cancer, confirming its involvement in the initiation and progression in gastric cancer and further suggesting a potential tumor‐suppressor role for miR‐302b in tumorigenesis of gastric tissue, which was consistent with previous studies 21, 22. Further analysis on the relation of miR‐302b expression with gastric cancer clinical parameters demonstrated that miR‐302b also had certain relation with lymph node metastasis, metastasis distance, and TNM stages, which strongly support our conclusion on the implication of miR‐302b in tumorigenesis of gastric tissue. To further explore the possible mechanism of miR‐302b in gastric cancer, our study also investigated the overexpression and inhibitor of miR‐302b on cell proliferation and cell cycle using SGC‐7901 cell line. Our results demonstrated that downregulation of miR‐302b can enhance the cell proliferation of gastric cancer cells. Similar results have also been concluded that miR‐302b was correlated with tumor differentiation and lymph node metastasis in esophageal squamous cell carcinoma, implying that low miR‐302b expression might be a poor prognostic factor 23.

Further mechanism analysis showed that *CDK2* was a target gene for miR‐302b. CDK2 is a key member of the CDK family, activation of cyclin E‐CDK2 complexes promotes cell cycle into the S phase through the G1/S restriction point. After entry into the S phase, CDK2 and cyclin A form a complex, which is involved in DNA replication and centrosome replication and regulation, promote mitosis occurs in G2/M conversion process 14. According to previous studies, the miR‐302‐367 cluster can regulate the expression of a number of target genes such as *CDK* to downregulate the expression of stem cells and related proteins 23, 24. In this study, after 48 h of miR‐302b overexpression, CDK2 regulation of miRNA and protein expression was significantly decreased, suggesting that miR‐302b may inhibit CDK2 in its regulation of the cell cycle. In addition, the possible role of ERK signal pathway was also verified on whether miR‐302b can regulate ERK signal pathway through CDK2. In this study, 48 h after transfection with miR‐302b, ERK levels were significantly decreased in gastric cancer cells, suggesting that miR‐302b may influence the development of gastric cancer via regulation of the ERK signaling pathway. The ERK is the first cloned and identified member of MAPK family. The ERK cascade primarily affects cell proliferation and differentiation 25. MEK is one of the main ERK pathways, MAPK1 (MEK1) phosphorylation activation can activate MAPK (ERK1/2) phosphorylation activation, and eventually activate the downstream cyclin and CDK protein to form a composite body, promoting the cell entering from G1 phase into S phase. ERK1/2 protein's activation in the phosphorylation process indicates that the changes in the signal pathway might be due to changes in the expression of phosphorylated ERK1/2, instead of ERK1/2 expression. Therefore, the miR‐302b accelerates the proliferation of gastric cancer cells by suppressing ERK1/2 phosphorylation 26, 27. It has been shown that, miR‐497 and other miRNA can inhibit cell cycle pathways through targeted regulation, in order to promote the development of cancer cells 28. Therefore, it is reasonable for us to speculate that upregulation of miR‐302b negatively targeting at *CDK2* can decrease cell proliferation and cell cycle transformation by inhibiting ERK signal pathway.

Our results showed that miR‐302b can target and negatively regulate the expression of CDK2 to decrease cell proliferation and arrest cell cycle in gastric cancer tissues via suppressing ERK signal pathway. However, miRNAs can regulate multiple target genes, and each gene can be regulated by different miRNAs 29. Therefore, other genes involved in the proliferation and metastasis of other tumors may also be targeted and regulated by miR‐302b. In addition, the sample does not have the capacity for investigation of these alternative pathways. Therefore, the mechanism of miR‐302b targeted regulation remains to be further confirmed in future studies.
